# Sir T. Lauder Brunton on Indigestion

**Published:** 1900-05-12

**Authors:** 


### SIR T. LAUDER BRUNTON ON INDIGESTION.

In a clinical lecture delivered a little time ago at St. Bartholomew's Hospital, Sir T. Lauder Brunton[1] laid down a series of rules as to the prevention and treatment of indigestion, which may be condensed as follows:—

The first part of digestion is mastication and insalivation, and if these things are not thoroughly performed, if the food is not perfectly comminuted by the teeth, and perfectly insalivated by the saliva, too great a strain is thrown upon the stomach. This is not merely a matter of slow eating. Many people who say they eat slowly still neither masticate thoroughly nor move the food about sufficiently in the mouth to ensure complete insalivation. "I have seen a man stick his fork into a whole new potato," says Sir Lauder Brunton, "and with a gulp and a gobble, but without any pretence of mastication, as the Americans say, 'get outside it.' Then he complained that new potatoes were indigestible." If a patient will not of his own accord follow the rule of completely masticating his food then he must count his bites; 32 bites to each mouthful of meat —one bite to each tooth—64 bites if it is tough, and 96 bites if it is very tough!

The next rule is that the patient should take his solids and liquids separately. For this there are two reasons; first, that if he swallows much liquid he dilutes his gastric juice, and thus lessens its digestive power, and second that one cannot get down unmasticated food without liquid, whereas, on the other hand, if one drinks during a meal one may chew very imperfectly, and wash the food down in lumps. At breakfast, the food at which is generally of a softer character, and chiefly farinaceous, one may allow a little more latitude, and the patient can take a breakfast-cupful of tea if he likes, but even then he should take it towards the end of the meal.

Then a third rule is to take farinaceous and proteid foods at different meals. Let the stomach do one thing at a time; take bread and butter for breakfast, but no fish, eggs, nor meat. In the middle of the day fish eggs, or meat may be taken, but no farinaceous food whatever. About five o'clock there should be a farinaceous meal, as at breakfast, and again at eight a proteid meal as at luncheon. If only food of the same kind is put into the stomach at each meal there is no delay from the different digestibility of the different kind of food, and the whole contents of the stomach become comminuted and digested, and passed on into the intestine in about the same time.

Coming back, however, to the question of fluid, it is clear that this must be taken—but not at meals. The best liquid the patient can drink is hot water, and the best times to drink it are on rising in the morning, again between eleven and twelve in the forenoon, again about four o'clock in the afternoon, and lastly at night before going to bed. In this way the patient gets all the fluid which he requires, not, however, at such times as to dilute the gastric juice when it is needed for digestion, but rather so as to assist in washing out of the stomach the remnants of the previous meal. In this way the stomach is kept clean for the succeeding meal. Certain medicines also are in some cases desirable. Where the stomach is weak some acid and pepsin may be given, and sometimes it is an advantage to give not pepsin alone, but pepsin containing rennin. Some of the essences of pepsin on the market appear to contain varying quantities of mixed enzymes, so that with one patient one preparation, and with another patient another kind, will be found beneficial. A little alkali also before meals is often advisable, as it tends to stimulate the secretion of gastric juice. Where there does not seem to be much catarrh of the stomach the alkali may be given with a bitter containing no tannin, such as calumba, but if there is much catarrh, better results are often obtained from bitters containing tannin such as gentian. Iron also may be of service, and where the tongue is large, pale, flabby, and marked by the teeth at the edges, better results are sometimes obtained from quassia and iron than from pepsin and other things which one might at first think would be better for the patient. Finally, when, as a result of taking large meals or drinking large quantities of water, more or less permanent dilatation of the stomach has taken place, and where the plan mentioned above of taking the solids and the fluids at different meals fails to give relief, the stomach may be washed out through a soft rubber tube either every morning just on rising or every night before going to bed.

[1] Clin. Jour., April 25.

